# Spontaneous Right Pneumothorax Manifesting as ST-Segment Elevation on ECG

**DOI:** 10.1016/j.jaccas.2025.106712

**Published:** 2026-01-19

**Authors:** Lei Liu, Tao Ye, Wenqi Tao, Panpan Xia

**Affiliations:** a Caojiadu Street Community Health Center, Shanghai, China; b Jing'an District Central Hospital, Shanghai, China

**Keywords:** electrocardiogram, right pneumothorax, ST-segment elevation

## Abstract

**Background:**

Electrocardiographic (ECG) findings in right-sided pneumothorax are clinically variable. This case details ECG changes characterized by ST-segment elevation in the high lateral leads, offering insights for clinical management, diagnosis, and treatment.

**Case Summary:**

A 63-year-old woman with spontaneous pneumothorax was admitted with severe cough and chest tightness. Her ECG revealed ST-segment elevation in leads I and aVL. Cardiogenic causes were excluded after further examination, attributing the symptoms to right-sided pneumothorax. The ECG normalized after the pneumothorax was resolved.

**Discussion:**

The ECG manifestations of right-sided pneumothorax are less clinically apparent compared with those of left-sided pneumothorax. This phenomenon is underpinned by multiple mechanisms, necessitating the integration of observed ECG findings to accurately localize the affected site and perform a comprehensive analysis.

**Take-Home Messages:**

The mechanism underlying right-sided pneumothorax is complex, with diverse clinical manifestations. Analyzing its etiology and tailoring treatment to the specific clinical context is essential.

## Visual Summary

**Figure:**
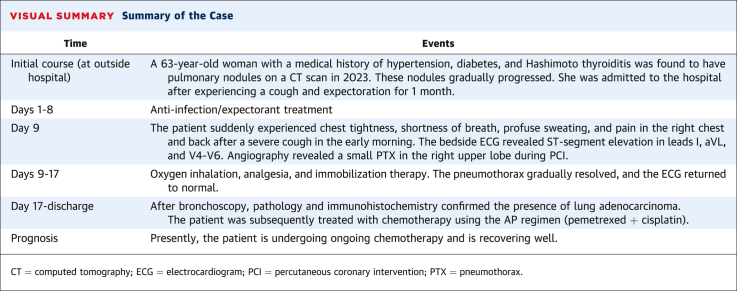

## History of Presentation

**Figure 1:**
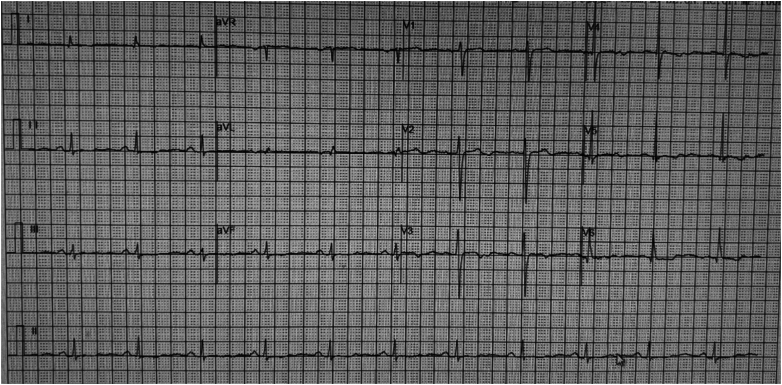
Electrocardiogram on Admission

**Figure 2:**
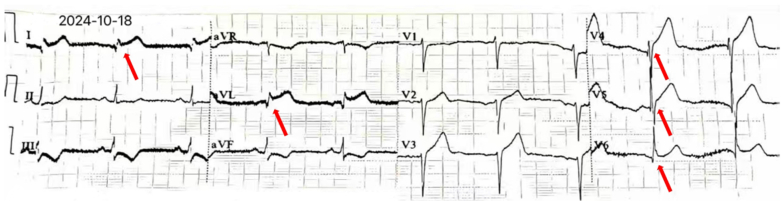
Electrocardiogram During an Episode of Chest Tightness After Severe Coughing Arrows indicate ST-segment elevation in leads I, aVL, and V_4_ to V_6_.

A 63-year-old woman presented with multiple nodular lung lesions on a 2023 chest computed tomography scan. Follow-up computed tomography in 2024 revealed disease progression, including a cavitary shadow in the right lower lobe. Upon admission on October 10, 2024, a further diagnostic work-up was performed. Blood tests showed a white blood cell count of 6.54 × 10^9^/L and hyperglycemia (fasting plasma glucose: 8.18 mmol/L). The electrocardiogram (ECG) showed sinus rhythm and flattened T waves in multiple leads (Figure 1); her cardiac biomarkers were normal (cardiac troponin T: 0.004 ng/mL, N-terminal pro–B-type natriuretic peptide: 116 pg/mL). Echocardiography revealed mild regurgitation of the mitral, tricuspid, and pulmonary valves, with a left ventricular ejection fraction of 61%. During hospitalization, the patient experienced an abrupt onset of severe cough without clear provocation, accompanied by chest tightness, dyspnea, and profuse sweating. The bedside ECG revealed ST-segment elevation in leads I, aVL, and V_4_ to V_6_, along with T-wave inversion in leads II, III, and aVF (Figure 2), suggestive of acute ST-segment elevation myocardial infarction. With the consent of the patient and her family, emergency coronary angiography was performed to assess potential coronary artery lesions.

**Take-Home Messages**

• The mechanism underlying right-sided pneumothorax is complex, with diverse clinical manifestations.
• Analyzing its etiology and tailoring treatment to the specific clinical context is essential.

## Medical History

The patient had a longstanding history of hypertension, diabetes mellitus, and Hashimoto thyroiditis. She consistently adhered to her medication regimen and reported well-controlled blood pressure and glucose levels, alongside normal thyroid function.

## Management

**Figure 3:**
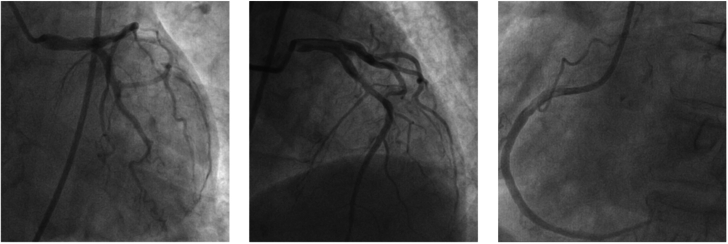
Coronary Angiography Examination No significant stenosis or myocardial bridging was observed on coronary angiography.

**Figure 4:**
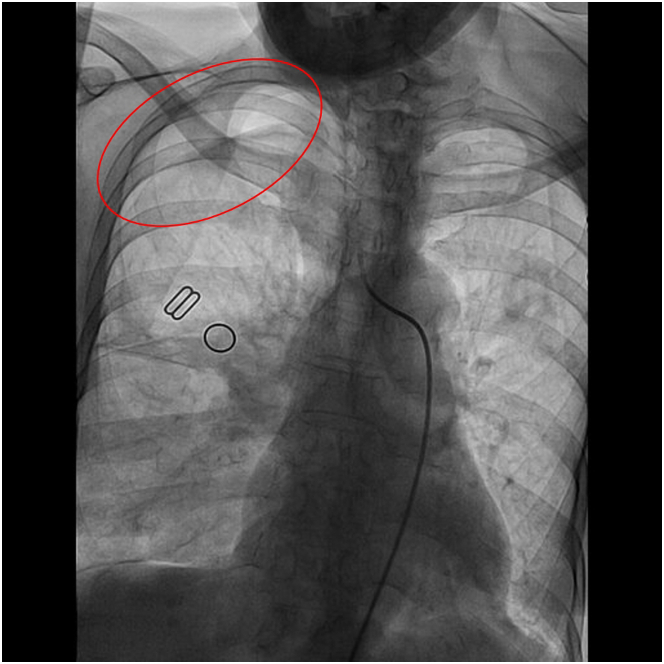
A Small Amount of Pneumothorax in the Right Upper Lung (Red Oval) Was Observed During Coronary Angiography

**Figure 5:**
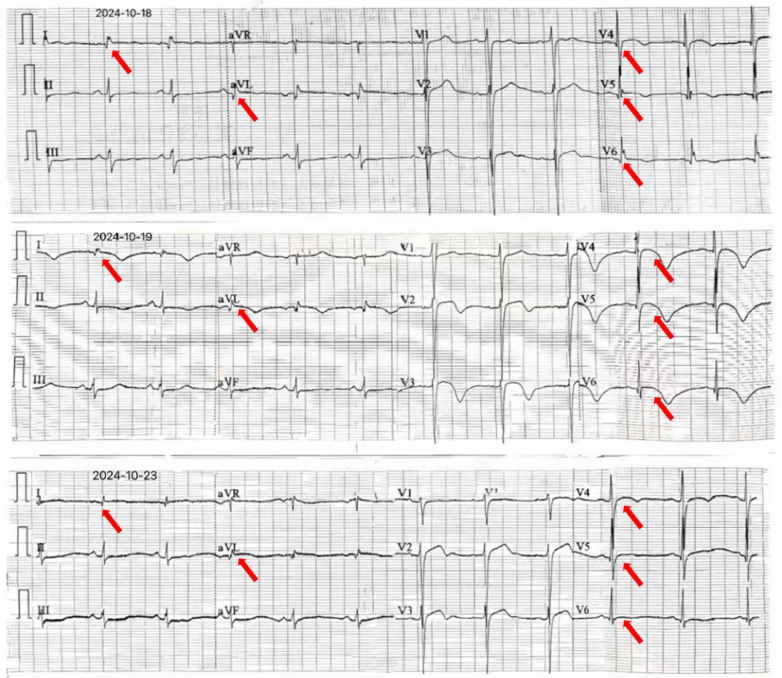
Dynamic Changes on Follow-Up Electrocardiogram The arrows indicate that the the pneumothorax gradually resolves, the electrocardiogram correspondingly returns to normal.

Angiography revealed no significant stenosis or myocardial bridging (Figure 3). Incidentally, a small pneumothorax (PTX) was detected in the right upper lobe (Figure 4). Oxygen therapy was administered, and serial ECG monitoring during the patient's hospital stay demonstrated dynamic changes (Figure 5).

## Outcome and Follow-Up

As the PTX was absorbed and the patient's condition improved, the ECG findings gradually normalized. These findings suggested that the acute myocardial infarction–like ECG manifestations in this patient were likely a consequence of the PTX rather than due to underlying coronary artery disease. After bronchoscopy, biopsy confirmed adenocarcinoma. The patient is receiving chemotherapy with the AP (pemetrexed and cisplatin) regimen and is recovering well. Chemotherapy is ongoing.

## Discussion

Our patient, with a history of lung disease, developed chest tightness after a severe cough. The ECG revealed ST-segment elevation in leads I, aVL and V_4_ to V_6_, mimicking acute myocardial infarction. However, coronary angiography showed no significant coronary artery abnormalities, and a small PTX was detected in the right upper lung during the intervention procedure. As the PTX resolved, the ECG changes gradually normalized. This suggested that the ECG findings, which resembled acute myocardial infarction, were likely attributable to the PTX.

Research indicates that around 25% of PTX patients display abnormal ECG findings.^1^ While left-sided PTX typically presents with specific ECG changes such as right QRS axis deviation, reduced QRS amplitude in precordial leads, ST-segment elevation, and T-wave inversion, the ECG manifestations of right-sided pneumothorax are less typical (Table 1). Yamamoto et al^1^ reported a case of severe right-sided PTX characterized by marked rightward deviation of the electrical axis on the ECG, with no evident ST-segment abnormalities in the limb and chest leads. This deviation may stem from the pressure exerted on the right atrium by the compressed right diaphragm during PTX, leading to axis deviation and amplitude alterations. Alzghoul et al^4^ reported a case of right-sided PTX showing ST-segment elevation in the anterior wall leads (V_1_-V_3_) after central venous line insertion. They attributed these transient ECG changes to gas accumulation in the thoracic cavity displacing the cardiac contour and exerting pressure on the heart and coronary vessels, potentially inducing ischemia. Pathophysiologically, PTX can induce hypotension, reduced venous return, decreased cardiac output, and tachycardia, exacerbating ischemia owing to heightened myocardial oxygen consumption. They noted fewer reports of ECG changes in right-sided PTX compared with left-sided cases, with less pronounced manifestations. Notably, significant ST-segment elevation in right-sided PTX was observed, with reversibility after pneumothorax resolution. Kataoka et al^5^ also described a case of right-sided PTX featuring ST-segment elevation in the inferior wall, likely linked to transient coronary ischemia from gas accumulation compressing coronary arteries in the pleural cavity.

**Table 1:** Differences Between Left-Sided and Right-Sided Pneumothorax

Classification	ECG Manifestations
Left-sided PTX	Right deviation of the QRS electrical axis, low voltage of the QRS complex, low voltage of the precordial R wave, ST-segment elevation, and T-wave inversion.^1^^,^^2^ These ECG manifestations may be attributed to the alteration of the anatomical position of the heart within the thoracic cavity and other potential mechanisms.^3^
Right-sided PTX	ST-segment elevation, right deviation of the electrical axis. (Compared with left-sided PTX, right-sided PTX lacks specific ECG manifestations.)

ECG = electrocardiogram; PTX = pneumothorax.

Reports on ECG manifestations of ST-segment elevation myocardial infarction in leads I and aVL due to PTX are scarce, and the underlying mechanisms remain unclear.^1^ In our case, ECG analysis showing ST-segment elevation in the lateral leads (I and aVL) and ST-segment depression in the inferior leads (II, III, and aVF) suggests transient compression of the intermediate or obtuse marginal branch.^6^ Potential contributing factors include 1) mechanical compression: Gas accumulation in the pleural cavity may compress the ventricles and coronary arteries, causing transient ST-segment elevation in specific leads. It is suspected that transient compression of the intermediate or obtuse marginal branch results in elevation in leads I and aVL, with normalization of the ECG after PTX resolution. 2) Changes in right-heart load: Compression of the right ventricle increases pulmonary artery pressure and decreases right ventricular compliance, resulting in clinical symptoms. 3) Hemodynamic changes: Elevated intrathoracic pressure impedes venous return and reduces cardiac output, causing low voltage in corresponding leads.

## Funding Support and Author Disclosures

The authors have reported that they have no relationships relevant to the contents of this paper to disclose.

**Visual Summary:** Summary of the Case

Time	Events
Initial course (at outside hospital)	A 63-year-old woman with a medical history of hypertension, diabetes, and Hashimoto thyroiditis was found to have pulmonary nodules on a CT scan in 2023. These nodules gradually progressed. She was admitted to the hospital after experiencing a cough and expectoration for 1 month.
Days 1-8	Anti-infection/expectorant treatment
Day 9	The patient suddenly experienced chest tightness, shortness of breath, profuse sweating, and pain in the right chest and back after a severe cough in the early morning. The bedside ECG revealed ST-segment elevation in leads I, aVL, and V4-V6. Angiography revealed a small PTX in the right upper lobe during PCI.
Days 9-17	Oxygen inhalation, analgesia, and immobilization therapy. The pneumothorax gradually resolved, and the ECG returned to normal.
Day 17-discharge	After bronchoscopy, pathology and immunohistochemistry confirmed the presence of lung adenocarcinoma. The patient was subsequently treated with chemotherapy using the AP regimen (pemetrexed + cisplatin).
Prognosis	Presently, the patient is undergoing ongoing chemotherapy and is recovering well.

CT = computed tomography; ECG = electrocardiogram; PCI = percutaneous coronary intervention; PTX = pneumothorax.
